# Effects of attachment security priming on women’s math performance

**DOI:** 10.3389/fpsyg.2023.1124308

**Published:** 2023-08-24

**Authors:** Antonio Soares De Almeida, Omri Gillath, Rotem Kahalon, Nurit Shnabel

**Affiliations:** ^1^Department of Psychology, University of Kansas, Lawrence, KS, United States; ^2^The Azrieli Faculty of Medicine, Bar-Ilan University, Safed, Israel; ^3^The School of Psychological Sciences, Tel Aviv University, Tel Aviv, Israel

**Keywords:** attachment security, priming, stereotype threat, domain identification, math performance

## Abstract

**Introduction:**

Activating people’s sense of attachment security can buffer against psychological threats. Here we tested whether security priming can also buffer the adverse effects of stereotype threat among women.

**Method:**

Three studies (a pilot study (*N* = 79 women, 72 men), a laboratory study; *N* = 474 women, and an online study; *N* = 827 women) compared security priming to neutral and positive affect priming.

**Results:**

The pilot study revealed that women exposed to attachment security primes (e.g., the word “love”) had better math performance than women exposed to neutral primes (e.g., “boat”). Men’s math performance did not differ across priming conditions. Study 1 revealed that women showed better math performance in the attachment security priming condition than in the neutral or positive (e.g., “luck”) priming conditions. The effect was observed among women high on math identification. In Study 2, despite an effect of security priming on the manipulation check [higher State Adult Attachment Measure (SAAM) security score], security did not buffer stereotype threat effects.

**Discussion:**

Our findings provide partial support to the idea that security priming (an interpersonal process) can buffer stereotype threat (an intergroup process). Theoretical and practical implications related to attachment security priming and stereotype threat are discussed.

## Introduction

Attachment theory is one of the most productive and interdisciplinary theories in psychology (for overviews, see Gillath et al., 2016; Cassidy and Shaver, 2018). According to the theory, people are equipped with an attachment system, which is activated when people encounter actual or symbolic threats in their environment. Once activated, the system motivates people to seek proximity to stronger wiser others termed “attachment figures”—often primary caregivers in infancy and childhood, and romantic partners/spouses or best friends in adulthood (Bowlby, 1969/1982). Being close to a supportive attachment figure makes people feel safe and secure, allowing them to regulate their emotions (“safe haven”) and explore the environment (“secure base”).

According to Ainsworth et al. (1978), people have different attachment styles—the way people think, feel, and behave in their close relationships. When a person’s attachment figures are consistently accessible and supportive, the person is likely to develop a *secure* attachment style. When attachment figures are not supportive, but instead are cold and rejecting, a person is more likely to develop an insecure *avoidant* style. Finally, when attachment figures are inconsistent (sometimes helpful and sometimes not) and intrusive in their caring, a person is more likely to develop an insecure *anxious* attachment style.

In the laboratory, researchers can make people feel more secure or insecure using attachment security primes. For example, researchers can expose participants to the names of their attachment figures or words such as “love” and “hug” (e.g., Gillath et al., 2010). Such priming can temporarily activate mental representations of attachment security, making participants feel, think, and behave like secure people (Gillath et al., 2022). Activating the sense of attachment security through exposure to security cues (security priming) increases people’s ability to constructively cope with internal and external stressors (Gillath et al., 2008) by buffering against threats (Gillath and Hart, 2010). For example, in one set of studies (Shaver and Mikulincer, 2007), participants were first exposed to either attachment security or neutral primes and then to either control or threat cues (e.g., threats to participants’ self-esteem or cultural worldviews). Exposure to threat cues led to a defensive derogation of outgroup members among participants in the neutral condition but not in the attachment security condition. Security primes did not affect the responses of participants who were not threatened, suggesting that activating attachment security may play an especially significant role in the face of threats (Mikulincer et al., 2001b).

Assuming that the attachment system helps coping with psychological threats, we tested whether exposure to attachment security primes would buffer against one specific psychological threat among women. Namely, *stereotype threat*—the state experienced by people who are, or feel themselves to be, at risk of conforming to the negative stereotypes about their social group (Steele, 1997).

### Stereotype threat among women

Stereotype threat may be conceptualized as a self-fulfilling prophecy, in which common negative perceptions about people’s social group “get under their skin” (see Goffman, 1963), leading to adverse psychological outcomes. For women, studying and being tested in STEM fields can be psychologically threatening due to the concern about confirming negative stereotypes regarding their gender’s math ability (Spencer et al., 2016). The experience of stereotype threat, in turn, might undermine the performance of women in math (see Walton and Spencer, 2009, for a meta-analysis). Admittedly, as a part of the replication crisis in social psychology (Open Science Collaboration, 2015), the magnitude of stereotype threat effects on women’s and girls’ math performance, and even the very existence of such effects, has been questioned (e.g., Ganley et al., 2013; Flore and Wicherts, 2015). Nevertheless, a non-zero effect such that women underperform when under threat (as compared to when not threatened) seems to be supported (Shewach et al., 2019)—justifying further research on this topic.

Existing research on stereotype threat has focused on providing an in-depth understanding of this phenomenon [e.g., Schmader et al.’s (2008) integrated process model] and on identifying interventions to reduce it. According to Liu et al.’s (2021) taxonomy, there are three types of interventions: identity-based interventions—which alter the strength or salience of one’s association with the negatively stereotyped ingroup, belief-based interventions—which change one’s belief(s) about the negative stereotype, and resilience-based interventions—which increase one’s ability to respond to the stressful situation in more adaptive ways by effectively regulating one’s emotions, approaching the task without self-defeating cognitions, or improving self-confidence.

We theorized that priming attachment security might serve as a resilience-based intervention, as it has been shown to improve people’s coping with psychological threats through changing their emotion regulation strategy use (e.g., Troyer and Greitemeyer, 2018), modulating intruding negative thoughts (e.g., Bryant and Chan, 2017), and promoting a more positive self-view (e.g., Carnelley and Rowe, 2007). Security priming also promotes self-confidence and autonomous exploration (Bowlby, 1969/1982). Therefore, we predicted that the exposure of women to attachment security cues would reduce the effects of stereotype threat on their math performance.

Our prediction is consistent with research showing that, due to their membership in a negatively stereotyped group, women become sensitive to situational cues that signal their inadequacy within potentially threatening situations (Murphy et al., 2007). Consequently, subtle situational cues have far-reaching effects on their thoughts, feelings, and performance. For example, the virtual classroom design (Cheryan et al., 2011) studied how stereotype threat affected women’s (but not men’s) interest and anticipated success in computer science. Thus, women’s level of interest and anticipation of success was lower in “geeky” classrooms (e.g., with Star Trek posters) as compared to non-geeky classrooms (Cheryan et al., 2011). Similarly, women’s (but not men’s) performance was affected by the gender composition of the group in which they took a math test. Specifically, their performance was impaired when outnumbered by men (Inzlicht and Ben-Zeev, 2000). While some situational cues increase the experience of stereotype threat, we reasoned that other situational cues might decrease it.

Theoretically, the present research seeks to extend attachment theory by integrating it with the stereotype threat literature. Research on attachment theory is highly diverse, and covers areas such as infant-parent relationships, social schemas, affect regulation, romantic love, marital functioning, group dynamics, prejudice, and intergroup relations (for a review, see Gillath et al., 2016). Nevertheless, the present research is the first to examine whether the attachment system may play a role in coping with a negatively stereotyped identity.

### The present research

A pilot and two studies investigate whether security primes can buffer stereotype threat effects. In the pilot, men and women were exposed to either security or neutral primes before taking a math test. We expected women, but not men, in the neutral priming condition to score lower on the math test in the presence of threat compared to women and men in the security priming condition. In Study 1, we compared the effects of attachment-security priming to positive priming to rule out the possibility that any positive prime would reduce stereotype threat effects. Another goal of Study 1 was to test the moderating effects of math identification. People who strongly identify with math (i.e., who perceived math as important and rewarding; Smith and White, 2001) are more vulnerable to stereotype threat since math is part of their self-concept (Schmader et al., 2008). High identifiers were expected to benefit more from the security priming than low identifiers. Finally, in Study 2, we used preregistration and a larger sample to increase confidence in our results. We expected participants who were exposed to stereotype threat and security priming to show less of a decrease in math performance than those who were exposed to threat and a neutral prime. We also expected the effects of the threat and the security priming to be most pronounced among participants who highly identify with math.

## Pilot study

We tested whether exposure to attachment security primes buffers the negative effects of stereotype threat on women’s (but not men’s) math performance using a 2 [participant’s gender (woman, man)] × 2 [prime (attachment security, neutral)] design. The dependent variable was the performance on a difficult math test. To avoid a potential conflation between participants’ gender and their knowledge of math (as more men than women take math-related majors in high school and higher education; Ayalon, 2003), our analysis controlled for participants’ preexisting knowledge of math.

### Method

#### Participants

Participants were 79 women and 72 men undergraduate students at a large Israeli university, *M*_*age*_ = 24.0, SD = 2.9, from various disciplines, whose native tongue was Hebrew. They received academic credit for their participation. The sample size was determined by feasibility considerations; data collection was stopped when there were no new sign-ups.

Whereas in earlier research on stereotype threat (e.g., Spencer et al., 1999) only women in math-related majors were recruited (because the assumption was that individuals must identify with the stereotyped domain to experience stereotype threat; e.g., Aronson et al., 1999), we examined a diverse sample, in line with subsequent research (e.g., Schmader, 2002; see Shapiro and Neuberg, 2007, for a discussion of stereotype threat effects among individuals who do not identify with a particular negatively stereotyped domain). More than half of the participants (38 women and 52 men) had math-related majors (e.g., engineering). The rest (41 women and 20 men) had majors that were not math-related (e.g., social sciences).

#### Procedure

The study, presented as “research on personality and academic performance,” consisted of two sessions. In the first session, participants individually completed an online survey, in which they reported their academic major (coded as “1” for math-related majors, and “0” for other majors), psychometric exam score [the Israeli equivalent of the Scholastic Aptitude Test (SAT) in the U.S. academic system], and their matriculation math exam’s difficulty level (“Yehidot Limod” ranging from three to five). Participants’ academic major, psychometric score, and math exam difficulty level were used as a proxy assessment of their preexisting math knowledge (e.g., acquaintance with relevant mathematical formulas). Participants also completed several background measures, such as the Experience in Close Relationship scale (ECR; Brennan et al., 1998), assessing attachment style along the dimensions of avoidance and anxiety.^1^ The full protocol and data are available via OSF: https://osf.io/2gd6y/?view_only=fcc001c569ed42d987303301526d73e7.

The second session was a laboratory experiment, carried out in groups of three to seventeen participants (depending on the sign-up) one week after the first session. About half of the participants in each session were women (52.3% female, SD = 18.5%). Participants first completed a priming task where they were asked to rate 20 pairs of furniture (e.g., “table” and “chair”) in terms of their degree of association on a 7-point scale (1 = *not at all* to 7 = *very much*). Participants were randomly assigned to one of two priming conditions such that before seeing each pair of furniture they were exposed either to neutral words (e.g., “boat,” “rug”) or to words, with matching length and frequency (in Hebrew), representing attachment security (e.g., “love,” “hug”). The prime was displayed in black lettering over a white background in the screen’s center for 22 ms, followed by a 500 ms mask (a visual noise pattern) and then by the names of the two pieces of furniture, separated by a hyphen (e.g., “cabinet-chair”). This task was successfully used before in various studies (e.g., Gillath et al., 2010).

Note that following the priming task, participants were asked whether they had noticed any words or letters that are not furniture pieces. More than half of the participants (63%) indicated that they had noticed such words or letters, and in most cases were able to generate at least one of the primed words. We included all participants because, according to Gillath et al. (2022), subliminal and supraliminal attachment security priming result in similar outcomes. Furthermore, our theorizing does not imply that the priming must be subliminal—we used this particular manipulation simply due to our wish to use a priming technique that was established in previous research.^2^

Following the priming task, participants completed a difficult math test composed of 30 multiple choice questions (see Kahalon et al., 2018, for the use of this test among Israeli women). To assure that the effects of the prime would last throughout the test after participants completed the first half of the test (20 min) they completed the priming task again. Participants then had additional 20 min to complete the second half of the test. Participants earned one point for each correct answer.

Next, participants completed the State Adult Attachment Measure (SAAM; Gillath et al., 2009). Consisting of 21 items and using a 7-point response scale. This measure captures situational fluctuations in people’s sense of attachment (in)security (e.g., “I feel like others care about me”). Note that the SAAM was developed such that security, avoidance, and anxiety should constitute different factors. However, because the differentiation between them was irrelevant for the purposes of the current study, we reverse coded the avoidance (e.g., “I’m afraid someone will want to get too close to me”) and anxiety (e.g., “I feel a strong need to be unconditionally loved right now”) items. This analytic approach is consistent with the theoretical conceptualization underlying the ECR coding (see Brennan et al., 1998), in which securely attached individuals are those low on the anxiety and avoidance dimensions. Then, participants’ responses were averaged to obtain a single SAAM score (α = 0.85).

This measure was used as a manipulation check, to verify that participants indeed felt greater security in the attachment security compared to the neutral prime condition. We did not administer the SAAM immediately after the priming manipulation—and before the math test—due to our concern that its items (e.g., “I feel loved”) might prime attachment security in both experimental conditions (by encouraging participants to think about their significant others) and thus interfere with the priming manipulation. Upon completion, participants were thanked and debriefed.

### Results and discussion

Means, SDs, and correlations are presented in Table 1. In line with previous reports (Ayalon, 2003), compared to women, men took more math-related majors (χ^2^ = 9.10, *p* = 0.003, *V* = 0.25) and more difficult math matriculation exams, *t*(149) = 2.14, *p* = 0.034, *d* = 0.68; and had higher psychometric scores, *t*(137) = 2.12, *p* = 0.040, *d* = 0.340. All three measures showed small to medium effect sizes, so the comparisons should be interpreted cautiously. To test our main hypothesis, we conducted a two-way analysis of covariance (ANCOVA) on participants’ math performance.^3^ The predictors were Gender (woman and man) and Prime type (security and neutral) and their interaction. Taking a math-related major, the difficulty of math matriculation exam, and psychometric scores were associated with participants’ math performance (see Table 1). We controlled for these variables to isolate the unique effects of priming condition on performance, which was the focus of the present inquiry (see Miyake et al., 2010, for the same analytic approach when testing the effects of a self-affirmation intervention on women’s math performance). To confirm randomization, the conditions were compared on attachment anxiety, attachment avoidance, psychometric score, math related major, math matriculation difficulty, and gender. No significant differences were found between the conditions. Results of this analysis are presented in the Supplementary material.

**TABLE 1:** Means, SDs, and correlations between the pilot study’s variables.

	Men		Women		(1)	(2)	(3)	(4)	(5)
	**Control**	**Security prime**	**Control**	**Security prime**					
1. Math related major	27 (79%)	25 (66%)	22 (56%)	16 (40%)	–	0.37**	0.57**	0.21+	0.37**
2. Psychometric score	686.88 (36.82)	686.29 (54.72)	673.90 (62.21)	658.56 (77.12)	0.15	–	0.62**	0.04	0.48**
3. Math matriculation difficulty	4.74 (0.51)	4.63 (0.63)	4.54 (0.76)	4.35 (0.77)	0.52**	0.42**	–	0.08	0.45**
4. Situational security	4.54 (0.72)	4.79 (0.83)	4.62 (0.73)	4.85 (0.58)	0.12	0.06	0.19	–	−0.01
5. Math score	15.47 (3.34)	14.66 (4.06)	13.10 (3.97)	14.00 (3.22)	0.22^+^	0.25*	0.33**	0.08	–

*N* = 79 women and 72 men students. The correlations for women are presented above the diagonal. ^+^*p* < 0.10, *p < 0.05, **p < 0.001.

As seen in Table 2, which presents the obtained model, the Gender × Prime type interaction was in the expected direction but only marginally significant.^4^ Importantly, women’s math performance was higher in the attachment security condition than in the neutral condition, *F*(1,144) = 4.02, *p* = 0.047, η^2^*_*p*_* = 0.03, 90% CI [0.001, 0.084].^5^ In particular, *M*_*difference*_ = 1.49, 95% CI [0.022, 2.967]. A sensitivity analysis [using Faul et al.’s (2009) calculator] revealed that for a 5% level of significance and a power of 80%, our actual sample size was sufficient to detect a minimum effect of *d* = 0.56; the observed effect size, *d* = 0.50, was somewhat below this minimum value.

**TABLE 2:** Results of two-way ANCOVA on math performance.

	Block I				Block II			
	**MS**	***F***	**Significant**	** $\eta_{p}^{2}$ **	**MS**	***F***	**Significant**	** $\eta_{p}^{2}$ **
Intercept	1.43	0.23	0.718	<0.00	2.43	0.23	0.635	<0.00
Psychometric score	82.89	7.57	0.007	0.05	88.86	8.26	0.005	0.05
Math matriculation difficulty	38.64	3.53	0.062	0.02	38.45	3.58	0.061	0.02
Math related major	23.71	2.17	0.143	0.02	23.64	2.20	0.140	0.02
Priming condition	9.64	0.88	0.350	0.01	8.00	0.74	0.390	0.01
Participant’s gender	16.15	1.47	0.227	0.01	17.38	1.62	0.206	0.01
Participant’s gender × priming condition					39.52	3.68	0.057	0.03

*N* = 79 women and 72 men.

**FIGURE 1:**
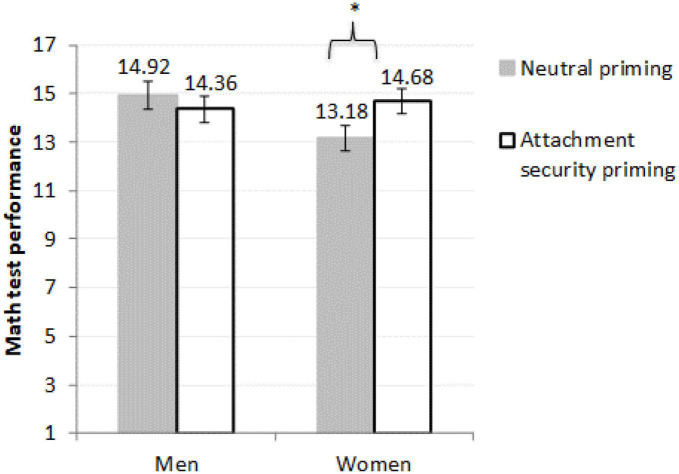
Mean number of correct answers and standard errors for math performance among women (*N* = 79) and men (*N* = 71) participants. Number of women = 79 and number of men = 71. Results are controlled for preexisting math knowledge. *Women in the secure priming condition scored significantly higher than women in the neutral condition [*F*(1,144) = 4.02, *p* = 0.047, η^2^*_*p*_* = 0.03].

Men’s performance did not differ across conditions, *F*(1,144) = 0.52, *p* = 0.473, η^2^*_*p*_* < 0.01, 90% CI [0.000, 0.037], *M*_*difference*_ = 0.560, 95% CI [−0.979, 2.098]. Figure 1 illustrates the obtained pattern of results (note that the means presented in this figure are corrected for the covariates and hence different than the raw means presented in Table 1).

As an alternative way to interpret this interaction, we compared women’s and men’s performance in each priming condition. We found that women performed worse than men in the control condition, *F*(1,144) = 4.93, *p* = 0.028, η^2^*_*p*_* = 0.03, 90% CI [0.002, 0.093], *M*_*difference*_ = 1.733, 95% CI [0.190, 3.276]. Yet, the gender gap was eliminated in the attachment security condition, *F*(1,144) = 0.18, *p* = 0.674, η^2^*_*p*_* = 0.001, 90% CI [0.000, 0.027], *M*_*difference*_ = 0.321, 95% CI [−1.828, 1.185]. This finding is consistent with the possibility that women experienced stereotype threat in the control condition (which led to the observed gender performance gap, evident even when controlling for preexisting knowledge in math), yet the exposure to attachment security primes in the experimental condition had buffered against stereotype threat effects—eliminating the gender gap in math performance.

As for the manipulation check (measured following the dependent variable), an ANOVA with participants’ SAAM score as the dependent variable revealed a significant difference between the Prime type conditions, *F*(1,147) = 4.35, *p* = 0.039, η^2^*_*p*_* = 0.028, 90% CI [0.001, 0.086], such that participants felt greater state security in the attachment security priming condition, *M* = 4.8, SD = 0.7, than in the neutral priming condition, *M* = 4.6, SD = 0.7. The effect of Gender and the Gender × Condition interaction were nonsignificant, *Fs* < 0.41, *p*s > 0.524, η^2^*_*p*_* < 0.003. These findings are consistent with the possibility that the priming manipulation induced participants with attachment security. Future studies should try to obtain bigger, more balanced groups of participants in each session if they are using the same method as we used here.

## Study 1

The pilot study provided initial support to our hypothesis that activating women’s attachment security can buffer the effects of stereotype threat on their math performance. Study 1 aimed to establish this effect using a sufficiently powered sample of women. It also aimed to demonstrate the uniqueness of this effect by testing whether women exposed to attachment security primes perform better not only compared to women exposed to neutral primes, but also to women exposed to positive affect primes that are unrelated to attachment security.

Performing a difficult math test might elicit negative thoughts and feelings among women (Keller and Dauenheimer, 2003; Cadinu et al., 2005). Possibly, the exposure to attachment security primes, which are more positive and pleasant than the neutral primes, simply elicited positive thoughts and feelings among women, leading to better math performance. The effects of attachment security primes, however, should stem from increasing one’s general sense of attachment safety (Mikulincer et al., 2005) rather than from the simple induction of positivity. To rule out positivity as an alternative explanation, women in Study 1 were exposed to attachment security-related primes (e.g., the word “love”), neutral primes (e.g., “lamp”), or positive primes unrelated to attachment (e.g., “luck,” “happiness”). These primes, which were matched in terms of length and language frequency (in Hebrew) and according to Mikulincer et al. (2001a) did not differ in terms of positive valence, were successfully used in previous research. We predicted that women’s math performance will be better in the attachment security priming condition than in the other priming conditions.

An additional goal of Study 1 was to explore the moderating role of domain identification. Women high in math identification suffer the most from stereotype threat both psychologically (Pronin et al., 2004) and in terms of their math performance (e.g., Keller, 2007; Kahalon et al., 2018). Thus, we tested whether the expected buffering effect of attachment security priming, compared to the other priming conditions, would be more pronounced among women who are high (vs. low) on math identification.

We tested our hypotheses among women using a three-cell experimental design [prime (attachment security, positive affect unrelated to attachment, and neutral)]. Our primary dependent variable was the performance on a math test. Consistent with the pilot study, to isolate the unique effect of the priming manipulation, we controlled for participants’ preexisting knowledge of math.

### Method

#### Participants

A power analysis using G*Power calculator (Faul et al., 2009) revealed that 368 participants were needed to detect a small effect size (*f* = 0.17, based on the size of the simple effect among women observed in the pilot study), at a significance of 5% and power of 80%. Note that the power analysis was conducted to detect the main effect of priming condition, not its moderation by math identification for which we had no a priori estimation. A sensitivity analysis indicated that our actual sample size was sufficient to detect a minimum interaction effect of *f*^2^ = 0.013. As reported below, the observed effect size, *f*^2^ = 0.010, was somewhat below this threshold. Participants were recruited through ads placed on campus and the university’s paid participant pool. They received 50 NIS (approximately 13 Euro or USD) in exchange for their participation. Data collection was stopped after the recruitment of 476 participants, once there were no new sign-ups. Participants were all women, undergraduate students, majoring in diverse disciplines (e.g., engineering, business); *M*_*age*_ = 23.6, SD = 2.7. All participants were Israeli, and their native tongue was Hebrew (17 bilingual).

#### Procedure

The procedure was similar to that of the pilot study, except that, to minimize attrition, participants completed it in a single session (instead of two separate sessions). Upon their arrival at the lab, participants reported their math identification on a four-item, 7-point scale (e.g., “I enjoy math and math-related fields”; α = 0.82), as well as their academic major (“1” for math-related majors, “0” for other majors), psychometric exam score, and matriculation math exam’s difficulty level, which served as proxy assessments of their preexisting math knowledge. They also completed several background measures (e.g., the ECR; see data in the OSF).^6^

Next, to strengthen the experience of stereotype threat, which in the pilot study was assumed to be induced through the presence of male participants, participants were told that as a part of the study they would be asked to complete a difficult math test, the results of which would be used to assess gender differences in math ability and develop the test norms for women and men (these threatening instructions were adjusted from Johns et al., 2005). Before proceeding to the math test, participants completed the priming task in which they rated the similarity between pairs of furniture (see section “Pilot study”). They were randomly assigned to one of three conditions: exposure to words representing attachment security (e.g., “love”), positive words unrelated to attachment (e.g., “luck”), and neutral words (i.e., “boat”).

Next, participants had 15 min to complete a math test that included 30 difficult questions. The Hebrew version of this test, developed by Johns et al. (2005), was validated and used by Kahalon et al. (2020). Participants earned one point for each correct answer. As a manipulation check for priming attachment security, participants completed the SAAM (α = 0.83). Upon completion, participants were thanked and debriefed.

### Results and discussion

Means, SDs, and correlations are presented in Table 3. To test our main hypothesis, we conducted a regression analysis on participants’ math performance. The covariates (taking a math-related major, math matriculation difficulty and psychometric scores; standardized) were entered in the first step. Prime type [attachment-security vs. control (positive unrelated to attachment and neutral primes)] and math identification (standardized) were entered in the second step, and their two-way interaction was entered in the third step. Randomization was tested by comparing the prime condition on attachment anxiety, attachment avoidance, domain identification, psychometric score, math related major, and math matriculation difficulty. Attachment anxiety and math difficulty are the only measures which differed between conditions.^7^ Results are presented in the Supplementary material. two additional analyses, in which (a) the attachment security priming condition is compared to each of the control conditions separately, (b) the neutral priming condition is compared to each of the positive priming conditions (i.e., attachment-related and unrelated) separately, are reported in the Supplementary material. Since the effects of the positive and neutral primes were not significantly different from each other, and security priming marginally differed from both control conditions, we aggregated the two control conditions to increase statistical power.

**TABLE 3:** Means, SDs, and correlations between the Study 1’s variables.

	Control (attachment-unrelated)		Security prime	(1)	(2)	(3)	(4)	(5)	(6)
	**Neutral**	**Positive words**							
1. Math related major	46 (36%)	32 (25%)	49 (39%)						
2. Psychometric score	672.8 (55.5)	670.6 (51.5)	680.9 (49.6)	0.29**	–				
3. Math matriculation difficulty	4.4 (0.7)	4.3 (0.7)	4.5 (0.6)	0.40**	0.39**	–			
4. Situational security	4.82 (0.6)	4.80 (0.8)	4.83 (0.6)	0.08	0.10*	0.11*	–		
5. Math identification	4.6 (1.5)	4.5 (1.4)	4.7 (1.6)	42**	0.30**	0.53**	0.01	–	
6. Math score	8.9 (4.2)	8.5 (3.4)	9.3 (4.1)	0.24**	0.50**	0.39**	0.05	0.36**	–

*N* = 474 women. *p < 0.05, **p < 0.001.

**FIGURE 2:**
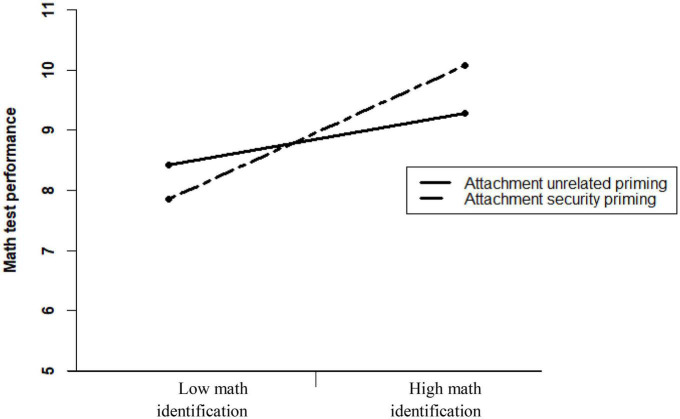
The effect of exposure to attachment security primes vs. control (neutral and positive) primes (*N* = 474) on math test performance among women whose math identification is high (+1 SD above average), *B* = 0.80 (SE = 0.45), *t* = 1.77, *p* = 0.077, vs. low (–1 SD), *B* = –0.59 (SE = 0.48), *t* = –1.23, *p* = 0.219.

As seen in Table 4, inconsistent with the effect observed in the pilot study, the effect of Prime type failed to reach significance, indicating that the math score of participants who were exposed to attachment security primes was not higher than that of participants who were not exposed to such primes. The Prime type × Math identification interaction was significant.^8^ The region of significance, calculated using Preacher et al.’s (2006) online calculator, was *Z*_*identification*_ > 1.50 at the higher bound, and *Z*_*identification*_ < −6.17 at the lower bound. Since the lower bound is practically meaningless, these results indicate that participants with relatively high math identification (whose standardized level of math identification was higher than 1.50) had better math performance if exposed to the attachment security primes rather than to neutral or positive primes that are unrelated to attachment security. Figure 2 illustrates the obtained results (note that because the slopes are presented for participants who are 1 SD above or below the mean, the slope for participants with high domain identification represents a non-significant trend).

**TABLE 4:** Results of regression analysis on math performance.

	Block I			Block II			Block III		
	***B***	***t***	**95% CI**	***B***	***t***	**95% CI**	***B***	***t***	**95% CI**
Constant	8.90	58.86***	[8.61, 9.20]	8.85	48.15***	[8.49, 9.21]	8.84	48.27***	[8.48, 9.20]
Math related major	0.14	0.86	[−0.19, 0.47]	<0.01	0.01	[−0.36, 0.34]	−0.01	−0.04	[−0.34, 0.33]
Math matriculation difficulty	0.85	4.90***	[0.51, 1.19]	0.60	3.18**	[0.23, 0.97]	0.60	3.17**	[0.23, 0.97]
Psychometric score	1.60	9.63***	[1.27, 1.93]	1.55	9.36***	[1.22, 1.88]	1.55	9.39***	[1.22, 1.87]
Priming condition (PR)				0.14	0.44	[−0.49, 0.77]	0.11	0.36	[−0.52, 0.73]
Math identification (MI)				0.61	3.29**	[0.25, 0.97]	0.43	2.09*	[0.03, 0.83]
PR × MI							0.69	2.05*	[0.03, 1.36]

*N* = 474 women. *p < 0.05, **p < 0.01, ***p < 0.001. Block I: *R* = 0.55, Δ*R*^2^ = 0.29, *F*(3,470) = 66.74, *p* < 0.001, Δ*F* = 66.74, *p* < 0.001. Block II: *R* = 0.56, Δ*R*^2^ = 0.02, *F*(2,468) = 43.01, *p* < 0.001, Δ*F* = 5.49, *p* = 0.004. Block III: *R* = 0.57, Δ*R*^2^ = 0.01, *F*(1,467) = 36.79, *p* < 0.001, Δ*F* = 4.21, *p* = 0.041.

As for the manipulation check, a one-way ANOVA, *F*(2,471) = 0.07, *p* = 0.931, η^2^*_*p*_* < 0.001, revealed that there was no significant difference between participants’ SAAM scores in the different priming conditions (see Table 3). Thus, inconsistent with the pilot study, we did not gain evidence that participants in the attachment security priming condition felt greater state attachment security than in the two control, attachment-unrelated conditions.

While unexpected, we believe that this result should be taken cautiously. As explained earlier, because of our concern that exposure to the SAAM’s items might induce attachment security and thus interfere with our manipulation, participants completed this measurement only after they completed the math test. By this time, the effect of the manipulation might have faded away. Previous research assumed that security had been successfully induced based on the effects of the ultimate outcome variables (e.g., outgroup derogation, Shaver and Mikulincer, 2007). We believe that a similar approach can be useful in the current study as well.

## Study 2

In Study 2 we wanted to replicate and extend the results of Study 1. Based on a power analysis (see details below) we recruited a large new sample to complete an online study. Study 1 partially supported the hypothesis that security primes improve math performance for women more than positive or neutral primes. To strengthen causal inference, stereotype threat was manipulated and compared to no threat in Study 2. Manipulating stereotype threat and comparing it to a no threat condition can help showing that security priming improves test performance specifically in the presence of threat. In light of the results of Study 1, and to increase power, the positive priming condition was not used in Study 2.

Women were recruited to complete an online survey about interpersonal relationships and problem-solving ability. They were exposed to either a threat or a neutral/control condition, followed by an attachment security prime (or control). Stereotype threat was induced via the instructions at the beginning of the study and again before the math test. In the threat condition participants were told we were testing math ability. In the control condition participants were told we were testing for problem-solving ability.

It was predicted that participants exposed to a stereotype threat will perform worse on the math test than participants in the non-threat condition (i.e., a main effect for the threat condition). It was further predicted that following exposure to a threat, participants in the security priming condition will perform better on the math test than participants in the neutral priming condition; the effect of security priming was expected to be smaller in the no-threat condition (i.e., a two-way Threat × Priming interaction). Finally, we predicted a three-way interaction between Threat, Priming, and domain identification. We expected that the buffering effect of security priming will be stronger among participants who were threatened and highly identify with math.

### Method

#### Participants

A power analysis using G*power was conducted to find the minimum sample size required to have a small effect size for the three-way interaction between priming, threat, and self-reported domain identification. The power analysis showed that ∼860 participants would be needed to detect a small effect size at a significance of 0.05 and power of 80%. Previous studies on stereotype threat were mainly done with participants physically present in the laboratory and often with the test done on paper (Shaffer et al., 2013; but see Schmader et al., 2004). We created an online version participants completed wherever they were, without coming to the lab, and using their own computer or phone. We expected these differences to increase variance among participants and reduce effect size (hence the bigger sample size). A total of (*N* = 960) participants were recruited via the online platform Prolific, which reached participants in the United States, the United Kingdom, and Australia (Prolific, 2014). Demographics are reported in the Supplementary material. They were compensated with $3.17 USD per participant. Preselection criteria were set to only allow participants who identified as female and native English speakers to take part. Of the 960 participants, 133 participants did not report their major, and were not included in the analysis. Participants’ ages ranged from 18 to 54 years old (*M*_*age*_ = 22.55, SD = 4.619).

#### Procedure

Participants accessed the study via a link to a survey on Qualtrics (2005). After consenting, participants were randomly assigned to one of the four experimental conditions. Based on the condition assignment they were exposed to either an attachment security and a stereotype threat prime (*n* = 210), security and no threat prime (*n* = 212), non-attachment-related neutral prime and threat (*n* = 203), or a neutral and no threat prime (*n* = 202). The analysis was preregistered^9^ and the data were posted^10^ on OSF. The four items using domain identification were averaged to create a value for each participant. An exploratory analysis which used a median split for domain identification is included in the Supplementary material.

##### Security/control priming tasks

Participants were exposed to the attachment security/neutral control primes via two tasks. The first task included exposure to either an attachment security-related image (an image of an elderly woman kissing an elderly man on the cheek) or a neutral image (a black and white image of a drink container partially in frame). The secure image was sourced from McGuire et al. (2018). To ensure the control prime was neutral, 40 participants evaluated six images on how happy, sad, secure, and neutral they made them feel, using a 4-point scale ranging from 1 (*not at all*) to 4 (*very much*). The four emotional states for each of the six images were compared using a 4 × 6 repeated measures ANOVA to identify the image lowest on the emotion measures and highest on neutrality. The main effects of image, *F*(35,5) = 8.76, *p* < 0.001, and emotion, *F*(35,3) = 18.136. *p* < 0.001, were significant as well as the interaction effect of image by emotion, *F*(35,15) = 8.968, *p* < 0.001. Pairwise comparisons revealed two images differing significantly from all others, one of which had low means for each of the emotionality measures and a high mean for neutrality (see Supplementary material). Participants had 2 min to observe the image and were instructed to remember as much detail as they could about it. Then they were asked to describe the image and how they felt when observing it.

In the second priming task, participants were given a list of eight sentences (three of which were always neutral) and had 2 min to memorize them. The sentences were either attachment-security-related (e.g., “John and Betty trust each other completely”) or control sentences (e.g., “The bookshelf was set up in the living room”). This task was successfully used by McGuire et al. (2018). Next participants were asked to write as many sentences as they could remember.

##### Threat vs. non-threat conditions

Participants were exposed to a stereotype threat during the informed consent and again in the instructions to the math test. The section of the informed consent read “We are conducting this study to better understand the links between interpersonal relationships and problem-solving skills/math ability” and the test instructions had “Once again, we ask you to do your best despite the difficulty, for the test to optimally reflect *your problem-solving skills/math ability*.” In the control condition, the study was described as assessing problem-solving ability, whereas in the threat condition, the study was described as assessing math ability.

##### Math performance

Math performance was assessed using ten math questions. The questions came from the same source as in Study 1. Participants were scored on how many questions they answered correctly.

##### Manipulation check

Nine items from the SAAM were used to measure state attachment avoidance, anxiety, and security. The three items measuring security (α = 0.79) were used to test the effectiveness of the primes by comparing the averages between priming conditions. The questions were administered after the math test to avoid the questions having a priming effect on math performance.

##### Demographics

Participants were asked to report their sex, age, ethnicity, education level, employment status, self-reported average high school math grade, and degree major. Majors were grouped into either STEM or non-STEM. STEM majors represented 44.7% of the participants.

### Results and discussion

Means, SDs, and correlations are presented in Tables 5 and 6. We first used a *t*-test to compare participants in the security priming condition with those in the neutral condition and found that participants exposed to security primes were significantly higher *t*(957) = 2.036, *p* = 0.042 on state attachment security than those in the neutral priming condition. These results are consistent with the pilot study, but inconsistent with Study 1. To test randomization, the conditions were compared on domain identification and STEM major. There were no significant differences between the conditions. The complete results are presented in the Supplementary material.

**TABLE 5:** Correlations between major, domain identification, and math performance in Study 2.

	1	2	3
1. Math-related major	–		
2. Domain identification	0.458**	–	
3. Math score	0.240**	0.288**	–

**Correlation is significant at the 0.01 level (two-tailed). Math related major was coded as 0 for non-STEM major and 1 for STEM major.

**TABLE 6:** Means and SDs of math performance in Study 2.

Prime condition	Math condition	Major	Domain identification	Mean score	SD	*N*
Neutral	No threat	Non-STEM	Low	4.26	2.07	80
			High	4.70	2.14	30
		STEM	Low	4.11	1.79	28
			High	6.03	1.77	64
	Threat	Non-STEM	Low	4.11	1.73	84
			High	5.67	2.31	21
		STEM	Low	4.79	1.90	33
			High	5.48	2.35	65
Secure	No threat	Non-STEM	Low	4.18	2.00	97
			High	4.81	2.08	27
		STEM	Low	4.96	2.14	23
			High	5.68	1.95	65
	Threat	Non-STEM	Low	4.28	2.29	90
			High	4.60	2.31	30
		STEM	Low	4.69	2.04	29
			High	5.75	1.89	61

A linear regression was used to analyze the effect of security priming (prime), stereotype threat (threat), and domain identification. Prior math knowledge (STEM major and non-STEM) was controlled in the first step. The main effect of prime, threat, and domain identification were entered in the second step, followed by the two-way interactions of each variable and finally the three-way interaction. Due to the number of variables and random assignment of conditions, some of the cell sizes were less than 30 participants. This reduced the power of the results and should be taken into consideration when interpreting them. The analysis revealed a main effect for previous math knowledge in the first step, *β* = 1.028, *t* = 7.090 *p* < 0.001, such that STEM majors scored higher than non-STEM majors. The analysis also revealed a main effect for Domain identification in the second step, *β* = 0.568, *t* = 8.870, *p* < 0.001, showing that the more a participant identifies with math, the higher they scored. The two and three-way interactions were not significant (see Table 7). So, although the security prime resulted in a higher SAAM security score, it did not seem to buffer the effects of stereotype threat as reflected in the regression.

**TABLE 7:** Results of regression analysis on math performance.

	Block I			Block II			Block III			Block IV		
	***B***	***t***	**95% CI**	***B***	***t***	**95% CI**	***B***	***t***	**95% CI**	***B***	***t***	**95% CI**
Constant	4.37	45.14	[4.18, 4.56]	3.06	15.18	[2.67, 3.46]	2.83	9.13	[2.22, 3.44]	2.80	8.12	[2.12, 3.48]
STEM major	1.03	7.09	[0.74, 1.31]	0.34*	2.13	[0.03, 0.652]	0.34*	2.12	[0.03, 0.65]	0.34*	2.13	[0.03, 0.65]
Prime condition				−0.05	−0.38	[−0.32, 0.22]	0.15	0.39	[−0.60, 0.89]	0.20	0.41	[−0.76, 1.16]
Threat condition				−0.01	−0.09	[−0.28, 0.26]	0.28	0.74	[−0.46, 1.02]	0.33	0.68	[−0.63, 1.30]
Math identification				0.57***	8.87	[0.44, 0.67]	0.65***	6.48	[0.45, 0.84]	0.66***	5.85	[0.44, 0.88]
Threat × math identification							−0.005	−0.02	[−0.55, 0.54]	−0.12	−0.17	[−1.50, 1.27]
Prime × math identification							−0.10	−0.89	[−0.32, 0.12]	−0.12	−0.76	[−0.43, 0.19]
Prime × threat							−0.07	−0.60	[−0.29, 0.15]	−0.09	−0.56	[−0.39, 0.22]
Prime × threat × math identification										0.04	0.17	[−0.40, 0.48]

*N* = 827 women. *p < 0.05, ***p < 0.001. Block I: *R* = 0.244, Δ*R*^2^ = 0.059, *F*(4,822) = 12.970, *p* < 0.001, Δ*F* = 12.97, *p* < 0.001. Block II: *R* = 0.375, Δ*R*^2^ = 0.141, *F*(3,819) = 19.182, *p* < 0.001, Δ*F* = 25.89, *p* < 0.001. Block III: *R* = 0.377, Δ*R*^2^ = 0.142, *F*(2,816) = 13.496, *p* < 0.001, Δ*F* = 0.34, *p* = 0.797. Block IV: *R* = 0.377, Δ*R*^2^ = 0.142, *F*(1,815) = 12.256, *p* < 0.001, Δ*F* = 0.01, *p* = 0.914.

In an additional exploratory analysis we performed, we dichotomized domain identification based on a median split. Prime type, threat, major, and categorical domain identification were entered as fixed factors, and education and self-reported average high school math grade were entered as covariates. The analysis revealed a main effect for domain identification, math grade, and major, and a marginal four-way interaction between prime type, threat, major, and domain identification (*p* = 0.076). Pairwise comparisons of the four-way interaction did not support a buffering effect of security priming on stereotype threat. Full results can be found in the Supplementary material.

## General discussion

The results of our studies produced mixed support for our research hypotheses. The pilot study revealed that women, but not men, in the attachment-security priming condition had better math performance than those in the neutral priming condition. Study 1 revealed that women in the attachment-security priming condition had better math performance than those in the control, attachment-unrelated conditions (either neutral or positive primes)—yet this effect was observed mainly among participants with high math identification. Although revealing priming effects, Study 2 did not replicate the results from the pilot study and Study 1. Thus, in a pilot and a lab study we showed small effects of security priming buffering threat effects on women in STEM (in Study 1: only among women who highly identify with math). When we ran the study online (during the pandemic) the stereotype threat results were not replicated. This inability to detect stereotype threat effects online is in line with other studies that did not replicate decreased math performance due to stereotype threat online (Finnigan and Corker, 2016; Kahalon et al., 2020). Although the study was bolstered by being preregistered and having a large sample, we did not find the expected buffering effect on stereotype threat (perhaps because there was no threat to buffer–no main effect for threat).

The findings of the pilot and Study 1 align with stereotype threat theorizing, according to which subtle cues that signal and create identity safety can eliminate the performance decrements caused by stereotype threat (Murphy and Taylor, 2012). Admittedly, whereas the manipulation checks in the pilot and Study 2 provided evidence that participants in the attachment-security priming condition indeed felt greater state attachment security than in the neutral priming condition, Study 1 did not provide such evidence. However, due to the reasons detailed in the discussion of Study 1, we believe this null effect does not hamper the main finding. The manipulation check in Study 2 showed a significantly higher state attachment security for participants in the security priming condition compared to the neutral priming condition. Compared to Study 1, the manipulation check took place closer to the priming in Study 2. This might have increased the chances of the the SAAM to detect the effects of the manipulation. The priming in the pilot study and Study 2 differed from the priming in Study 1 by not explicitly mentioning gender. Conceivably, the explicit mention of gender in Study 1 could have resulted in female participants feeling motivated to overcome the stereotype.

Theoretically, the inconsistent results of our research were not able to shed light on the potential interplay between individual-level and group-level processes. One’s sense of attachment security failed to provide a consistent “symbolic shield” that could help coping with social-psychological stressors such as negative stereotyping. Within attachment theory, previous research on the interplay between the individual and group levels has focused on the positive impact of attachment security on the *sources* of outgroup prejudice and stereotyping (Shaver and Mikulincer, 2007; Boag and Carnelley, 2016). The present research is the first to examine the potential impact of attachment security on the *targets* of these negative stereotypes. Specifically, we looked at the negative stereotype imposed on female about math performance. Although we expected security to buffer the negative effects of exposure to stereotype threat (decreased performance), we only obtained partial support.

### Practical implications considering the effect size

Our purpose was not just to enhance theoretical understanding but also to develop a simple strategy to improve women’s math performance in real-life settings. We reasoned that priming attachment security can be an ideal candidate for scaling up an intervention to improve women’s math performance due to its cost-effectiveness and easiness of administration—two aspects that are crucial for the scaling up of interventions to improve academic performance (Bakker et al., 2019; Kraft, 2019; see McGuire et al., 2018, for a discussion of the implementation of attachment security priming interventions in other contexts).

The effects in Study 1, however, were observed mainly among women relatively high on domain identification. Furthermore, the effects were relatively small (see raw means of participants’ math scores; in Tables 1, 3). Previous theorizing argued that a small effect observed in the lab may translate into a larger effect in a real-life intervention because academic settings create self-fulfilling cycles; namely, recursive processes in which initial psychological threat impairs performance, which further increases threat and yields consequent performance impairment (Cohen et al., 2009). It is as likely, however, that a small effect observed in the lab would disappear when tested in a noisy real-life context due to the interference of unobserved variables that were not considered when setting out a controlled lab study (Paluck and Cialdini, 2014). Our conclusion, therefore, is that priming attachment security might not be the best intervention to reduce gender gaps in math performance. More broadly, our research suggests that a single dose of security priming was not enough to reduce group-based disparities (Singal, 2021; see for example, Hanselman et al., 2017, for the fragility of widely implemented wise interventions based on self-affirmation exercises in reducing group-based achievements gaps).

Our findings also highlight the need to pay greater attention to effect sizes (Cumming, 2013). Some of the intervention studies that inspired the present research have focused solely on significance tests without discussing effect sizes. For example, Miyake et al. (2010) did not report the effect size for the improvement in physics grades among women in the self-affirmation compared to the control condition. Yet, in light of the debate about the replicability and validity of stereotype threat (e.g., Flore and Wicherts, 2015; Zigerell, 2017; Shewach et al., 2019) and priming effects (Doyen et al., 2012), effect sizes should always be discussed in research about these topics, especially when potentially practical interventions are tested.

### Limitations and future directions

Besides the failure to identify a “wise intervention,” a major limitation of the present research is that the studies (except for Study 2) were not pre-registered, which would strengthen the credibility of the conclusions (Nosek et al., 2018). As mentioned previously, employing stereotype threat manipulations online may have hindered the ability to detect a buffering effect. Future studies conducted in person may help disentangle the reason for our failure to detect effects (is it the online mode or security priming inability to buffer). Another limitation is that we focused on only one outcome, math performance. Future research can examine whether the activation of women’s attachment security influences other psychological outcomes of stereotype threat, such as women’s sense of misfit and inauthenticity in STEM environments (Schmader and Sedikides, 2018).

Future research could also test the impact of attachment security primes on stereotype threat among other minority group members. We suspect that the reason for the small effect observed in the present research is that besides activating attachment security, the primes (e.g., words like “hug”) unintendedly activated positive stereotypes about women’s nurturance and communality (which are related to women’s traditional roles as wives and mothers). Recent research (Kahalon et al., 2018, 2020) revealed that, despite their positivity, the activation of such stereotypes can lead to stereotype threat effects, resulting in women’s impaired math performance.

Examining the effects of attachment security priming on the reduction of stereotype threat effects among members of other negatively stereotyped groups, which are not stereotypically perceived as nurturing (e.g., African Americans performing an intelligence test; Steele, 1997), would possibly yield larger effects. Theoretically, attachment security serves two key functions: promoting a “safe haven” (i.e., relaxation) as well as a “secure base” (i.e., mobilization of energy to effectively cope with stressors). If so, then the positive effects of priming attachment security on performance should be particularly pronounced when there is “a threat in the air” (Ai et al., 2020), as is the case when African Americans take a test said to be diagnostic of intelligence (Steele, 1997).

Another direction future research should consider is using more personalized primes or repeated priming. Having a more personalized prime may increase the activation of the security schema, resulting in stronger effects. Repeated priming would further test the potential for security priming to be used as a wise intervention. The use of repeated primes may result in longer lasting effects, compared to the more transient nature of a single priming session (Gillath et al., 2008).

## Conclusion

The current work provides partial support for the effect of security priming on math performance under stereotype threat. In the lab, attachment security was shown to buffer the negative effects of stereotype threat, mainly among those who identify strongly with the domain. However, in a preregistered online study with a large sample, security priming did not buffer the negative effects of stereotype threat on women. The mixed results may have been due to modality differences (offline vs. online) or the presence of men vs. manipulating threat vs. suggested threat. Future work should look into identifying factors that could enhance the potential impact of security priming on stereotype threat and bridge the two bodies of research on intrapersonal and intergroup processes.

## Data availability statement

The original contributions presented in this study are included in the article/Supplementary material, further inquiries can be directed to the corresponding author.

## Ethics statement

The studies involving humans were approved by the University of Kansas Internal Review Board. The studies were conducted in accordance with the local legislation and institutional requirements. The participants provided their written informed consent to participate in this study.

## Author contributions

AS, OG, RK, and NS involved in deriving the hypotheses based on the literature, planning the experiments to test these hypotheses, analyzing the results, and writing the manuscript. All authors contributed to the article and approved the submitted version.

## Footnotes

1 The ECR was not included in the final analysis as neither main effect, attachment anxiety or avoidance, nor the interaction of attachment style and condition were significant. Full results can be found in Supplementary material.

2 The statistical conclusions reported in section “Results” do not change when controlling for whether or not participants noticed words or letters that were not furniture pieces.

3 It could be interesting to test whether the effects of security priming depended on the gender composition in each session using multilevel modeling. Due to one experimental session containing only women, some sessions not containing both priming conditions, and the small sizes of some groups, we did not employ multilevel modeling to evaluate group-level effects.

4 We believe that entering the three variables that were used as a proxy assessment of participants’ preexisting math knowledge (i.e., participants’ academic major, psychometric score, and matriculation math exam’s difficulty level) as covariates is justified because they all significantly correlated with participants’ math test score (see Table 1). Nevertheless, once entered *together* into the regression model, only psychometric score significantly predicted participants’ math test score (see Table 2). When repeating the regression analysis reported in Table 2 with only one covariate (i.e., participants’ psychometric score), the Gender × Prime type interaction remained marginal, *F*(1,146) = 3.50, *p* = 0.063. When repeating the regression analysis reported in Table 2 with two covariates (i.e., participants’ psychometric score and matriculation math exam’s difficulty level, which marginally predicted participants’ test score), the Gender × Prime type interaction also remained marginal, *F*(1,145) = 3.65, *p* = 0.058. In addition, because participants took the test in groups, it made sense to control for the variance in performance associated with experimental session – as lower ratios of women in a given session likely increased stereotype threat (Murphy et al., 2007). When adding it as a covariate to the model reported in Table 2, the effect of experimental session was significant, *F*(1,143) = 3.93, *p* = 0.049, and the Gender × Prime type interaction became significant, *F*(1,142) = 5.40, *p* = 0.022.

5 See Steiger’s (2004) explanation of why using 90% CI around η^2^*_*p*_* (which can only be positive), equals to 95% around Cohen’s *d* (which can be either positive or negative) for the same test.

6 The ECR was not included in the final analysis as neither main effect, attachment anxiety or avoidance, nor the interaction of attachment style and condition were significant. See Supplementary material for the complete results.

7 Using normalized attachment anxiety and math difficulty as covariates did not change the direction or significance of the results, so they are not included in the main analysis.

8 We believe that entering the three variables that were used as a proxy assessment of participants’ preexisting math knowledge (i.e., participants’ academic major, psychometric score, and math exam’s difficulty level) as covariates is justified because they all significantly correlated with participants’ math test score (see Table 3). Nevertheless, once entered *together* into the regression model, only psychometric score and matriculation math exam’s difficulty significantly predicted participants’ math test score (see Table 4). When repeating the regression analysis reported in Table 4 with only two covariates (i.e., participants’ psychometric score and matriculation math exam’s difficulty), the Math Identification × Prime type (Attachment security vs. other) interaction remained significant, *B* = 0.69, *t* = 2.05, *p* = 0.041.

9
https://doi.org/10.17605/OSF.IO/PW3KC

10
https://osf.io/t89ys/files/osfstorage/639a6f1ec0ae63000ec3ef6c
