# Supplementary material for: Effects of attachment security priming on women’s math performance
Source: Front Psychol. 2023 Aug 24;14:1124308. doi: 10.3389/fpsyg.2023.1124308 (PMC10484519; doi:10.3389/fpsyg.2023.1124308)
Supplement: Supplementary file 1 [file Table_1.docx]

**Supplemental materials**

**Baseline comparison of conditions in Pilot Study**

Supplementary table 1 contains the t-tests performed to compare the conditions on attachment anxiety, attachment avoidance, and psychometric score. There were no significant differences between the conditions on attachment anxiety (*p* = .909), attachment avoidance (*p* = .193), or psychometric score (*p* = .428).

Supplementary table 2 presents a Chi-squared test comparing the number of math related majors in each condition. There was not a significant difference in the number of math related majors (*p* = .068).

Supplementary table 3 shows the results of a Chi-squared test comparing the math matriculation difficulty by condition. There was not a significant difference in the math matriculation difficulty between conditions (*p* = .350).

Supplementary table 4 compares the gender composition of the conditions. There were not significant differences in the number of males and female between conditions (*p* = .792).

Supplementary table 5 contains the results of an additional analysis that was identical to what was reported in the paper except it controlled for attachment anxiety and attachment avoidance measured by the ECR. Previous work suggests attachment interventions such as security priming have a greater effect on those who are high on avoidance or anxiety (Cassidy et al. 2017). A recent meta-analysis showed the effects of priming was not affected by individual attachment style (Gillath et al. 2022). In an additional analysis, neither attachment anxiety nor avoidance moderated the effects.

**Baseline comparison of conditions in Study 1**

Supplementary table 6 contains the results of t-tests comparing conditions on psychometric score, domain identification, attachment anxiety, and attachment avoidance. Psychometric scores (*p* = .071), domain identification (*p* = .307), and attachment avoidance (*p* = .763) did not differ between conditions. Attachment anxiety was significantly higher in the attachment unrelated priming condition compared to the attachment security priming condition (*p* = .012).

Supplementary table 7 presents a Chi-squared test comparing the number of math related majors between conditions. There was not a significant difference in the number of math majors between the conditions (*p* = .142).

Supplementary table 8 shows the results of a Chi-squared test comparing the number of high and low identifying participants in each condition. There was not a significant difference in the number of high and low identifying participants between conditions (*p* = .456).

Supplementary table 9 presents a Chi-squared test comparing the math matriculation difficulty by condition. There was a significant difference in the math matriculation difficulty between conditions such that there were a greater number of participants who had a difficulty level of 2 than 1, and a greater number of participants with a difficultly number 3 than 2 and 1 (*p* = .025).

**Additional analyses: Study 1**

Supplementary table 10 presents a regression model similar to that presented in Table 4, except that the Priming condition was coded as two dummy-variables, in which the Neutral condition served as the reference category. As can be seen in the table, the Attachment security (vs. Neutral) × math identification interaction was marginally significant (*p* = .062), whereas the Positive unrelated to attachment (vs. Neutral) × math identification interaction was non-significant (*p* = .972). Preacher et al.’s (2006) online calculator was used to explore the marginally significant interaction of attachment security (vs. Neutral) × math identification. As can be seen in supplementary figure 1, the results are very similar to what is presented in the main analysis. High identifying participants showed greater performance in the secure condition compared to the neutral condition.

Supplementary table 11 presents a regression model similar to that presented in Table 4, except that the Priming condition was coded as two dummy-variables, in which the Attachment security condition served as the reference category. As can be seen in the table, the Neutral (vs. Attachment security) × math identification interaction was marginally significant (*p* = .062), and so was the Positive unrelated to attachment (vs. Attachment security) × math identification interaction (*p* = .079). The neutral (vs. Attachment security) × math identification interaction is an identical comparison to supplementary figure 1. We explored the Positive unrelated to attachment (vs. Attachment security) × math identification interaction in the same way. Supplementary figure 2 presents the same trends as the neutral vs. security comparison, and what is shown in the main analysis. High identifiers see a buffering effect from attachment security priming when compared to positive priming unrelated to attachment. Observing the same trends when comparing all three primes and when merging prime and neutral priming lends support towards combining the conditions.

Supplementary table 12 contains a regression model similar to the main analysis, but it includes the covariates attachment anxiety and attachment avoidance measured by the ECR. The analysis showed that neither attachment anxiety nor avoidance moderated the effect of security priming.

**Baseline comparison of conditions in Study 2**

Supplementary table 13 shows the results of an ANOVA comparing the conditions Prime [attachment security, neutral] and Math test [Stereotype threat, no threat] on domain identification. There were no significant differences between the conditions.

Supplementary table 14 contains the results of a Chi-squared test comparing the number of STEM and non-STEM majors in each condition. The four conditions resulting from the combination of Prime and Math test were combined to perform a 2x4 contingency test. There was not a significant difference in STEM and non-STEM majors between the conditions (*p* = .489).

**Additional analyses: Study 2**

Supplementary table 15 presents the mean ratings on four emotions for each of the six images tested for the control prime in Study 2.

Supplementary table 16 contains the demographics of the online sample collected for Study 2.

An exploratory analysis treated domain identification as a categorical variable. The four items measuring domain identification were averaged. An ANOVA tested the effects security-priming (prime), stereotype threat (threat), major (stem, no stem), and math identification (high, low) had on math test performance. Participant’s level of education and self-reported average high school math grade were included as covariates^[[1]](#footnote-1)^. To create high and low math identification, the four items were averaged. Participants at or above the median (Median = 3.00) were categorized as high on identification, and participants below the median were categorized as low. The analysis revealed a main effect for self-reported average high school math grade, identification, and major (people who had better grades, were high on identification, and those who reported majoring in stem had higher math scores). A main effect for education was marginally significant along with a four-way interaction between prime, threat, major, and identification, *F* (1, 672) = 12.105, *P* = .076, η² = .005. There were no other main effects or interactions (see Supplementary table 17).

Pairwise comparisons show the significant differences to be between high and low identifiers when exposed to neutral (*p* = .006) or secure (*p* = .020) priming, no stereotype threat, and are STEM majors. High and low identifiers also differed for non-STEM majors who were exposed to neutral priming and stereotype threat (*p* = .016). Additionally, high and low identifiers that are STEM majors exposed to security priming and stereotype threat differed (*p* = .044). In each case, high identifiers outperformed low identifiers, consistent with the main effect observed. Finally, a significant pairwise comparison found a difference between STEM and non-STEM majors who are high identifiers and were in the secure prime and threat conditions. STEM majors perform better on the math test than low identifiers (*p* = .029). It is apparent that the marginal significance of the four-way interaction is driven by these comparisons, which do not systematically differ across conditions in a way that conclusions can be drawn. With the ineffective online stereotype threat, Study 2 is largely inconclusive, and does not provide support for the hypotheses.

| *Comparison of baseline differences between conditions* | | | | |
| --- | --- | --- | --- | --- |
|  | t | df | *p* | Cohen’s d |
| ECR Avoidance | 1.308 | 149 | .193 | 0.213 |
| ECR Anxiety | .114 | 149 | .909 | 0.019 |
| Psychometric Score | .795 | 149 | .428 | 0.129 |

**Supplementary Table** **1**

*Note.* t-statistic was calculated as control minus security condition.

| *STEM and non-STEM majors by condition* | | | Major | | Total |
| --- | --- | --- | --- | --- | --- |
|  |  |  | Non-STEM | STEM |  |
| Condition | Control | Count | 24 | 49 | 73 |
|  |  | % | 32.9% | 67.1% | 48.3% |
|  | Security | Count | 37 | 41 | 78 |
|  |  | % | 47.4% | 52.6% | 51.7% |
| Total | | Count | 61 | 90 | 151 |
|  |  | % | 40.4% | 59.6% |  |

**Supplementary Table 2**

X^2^(1) = 3.320, *p* = .068, V = 0.148

**Supplementary Table** **3**

| *Condition * Math Difficulty Crosstabulation* | | | Math Difficulty | | | Total |
| --- | --- | --- | --- | --- | --- | --- |
|  |  |  | 3.00 | 4.00 | 5.00 |  |
| Condition | Control | Count | 7 | 13 | 53 | 73 |
|  |  | % within Condition | 9.6% | 17.8% | 72.6% | 48.3% |
|  | Security | Count | 10 | 20 | 48 | 78 |
|  |  | % within Condition | 12.8% | 25.6% | 61.5% | 51.7% |
| Total | | Count | 17 | 33 | 101 | 151 |
|  |  | % within Condition | 11.3% | 21.9% | 66.9% |  |

X^2^(2)= 2.099, *p* = .350, V = 0.118

**Supplementary Table** **4**

| *Gender by condition* | | | Gender | | Total |
| --- | --- | --- | --- | --- | --- |
|  |  |  | Male | Female |  |
| Condition | Control | Count | 34 | 39 | 73 |
|  |  | % within Condition | 46.6% | 53.4% | 48.3% |
|  | Security | Count | 38 | 40 | 78 |
|  |  | % within Condition | 48.7% | 51.3% | 51.7% |
| Total | | Count | 72 | 79 | 151 |
|  |  | % within Condition | 47.7% | 52.3% |  |

X^2^(1)= 0.069 , *p* = .792, V = 0.021

**Supplementary Table** **5**

| Source | df | MS | F | *p* | Effect size |
| --- | --- | --- | --- | --- | --- |
| Corrected Model | 8 | 69.30 | 6.40 | <.001 | .27 |
| Intercept | 1 | .18 | .02 | .90 | .000 |
| ECR_AVO | 1 | .30 | .03 | .87 | .000 |
| ECR_ANX | 1 | 10.69 | .99 | .32 | .007 |
| Psychometric Score | 1 | 89.51 | 8.27 | .005 | .06 |
| Math Diffuclty | 1 | 38.26 | 3.54 | .06 | .02 |
| Math-Related Measure | 1 | 18.03 | 1.67 | .20 | .01 |
| Condition | 1 | 6.82 | .63 | .43 | .004 |
| Gender | 1 | 17.61 | 1.63 | .20 | .01 |
| Condition X Gender | 1 | 39.71 | 3.67 | .06 | .03 |
| Error | 142 | 10.82 |  |  |  |
| Total | 151 |  |  |  |  |
| Corrected Total | 150 |  |  |  |  |

*Note*. MS = Mean squares, effect size = *η2* or partial *η2.* Independent variables were priming condition [neutral, secure] and Gender [Male, Female].

**Supplementary Table** **6**

| *Mean comparison of baseline differences by condition* | | | | | | | | |  |
| --- | --- | --- | --- | --- | --- | --- | --- | --- | --- |
|  | | t | df | | *p* | | Cohen’s d | |  |
| psychometric | -1.809 | | | 472 | | 0.071 | | -0.176 | |
| Domain ID | -1.022 | | | 472 | | 0.307 | | -0.186 | |
| ECR Avoidance | -.301 | | | 472 | | 0.763 | | -0.029 | |
| ECR Anxiety | 2.514 | | | 472 | | 0.012 | | 0.245 | |

*Note.* t-statistic was calculated as attachment unrelated priming minus attachment security priming

**Supplementary Table** **7**

| *Condition * Math Major Crosstabulation* | | Math Major | | Total |
| --- | --- | --- | --- | --- |
|  |  | Math related major | Other major |  |
| Prime | Attachment unrelated priming | 238 | 78 | 316 |
|  | Attachment security priming | 109 | 49 | 158 |
| Total | | 347 | 127 | 474 |

X^2^(1) = 2.151, *p* = 0.142, Cramer’s V = 0.067

| *Condition * Domain ID (high/low) Crosstabulation* | | Domain ID | | Total |
| --- | --- | --- | --- | --- |
|  |  | Low math identification | High math identification |  |
| Condition | Attachment unrelated priming | 253 | 63 | 316 |
|  | Attachment security priming | 131 | 27 | 158 |
| Total | | 384 | 90 | 474 |

**Supplementary Table** **8**

X^2^(1) = 0.555, *p* = 0.456, Cramer’s V = 0.034

**Supplementary Table** **9**

| *Condition * Math Difficulty Crosstabulation* | | Math Difficulty | | | Total |
| --- | --- | --- | --- | --- | --- |
|  |  | 1 | 2 | 3 |  |
| Condition | Attachment unrelated priming | 50 | 105 | 161 | 316 |
|  | Attachment security priming | 11 | 57 | 90 | 158 |
| Total | | 61 | 162 | 251 | 474 |

X^2^(2) = 7.395, *p* = 0.025, Cramer’s V = 0.125

| **Supplementary Table** **10**  *Results of Regression Analysis on Math Performance, with the Neutral condition as the reference category.* | | | | | | | | | |
| --- | --- | --- | --- | --- | --- | --- | --- | --- | --- |
|  | **Block ǀ** | | | **Block ǀǀ** | | | **Block ǀǀǀ** | | |
|  | *B* | *t* | *Sig.* | *B* | *t* | *Sig.* | *B* | *t* | *Sig.* |
| Constant | 8.90 | 58.86 | .000 | 8.93 | 34.68 | .000 | 8.93 | 34.76 | .000 |
| Math related major | .14 | .86 | .391 | -.00 | -.02 | .981 | -.01 | -.07 | .943 |
| Math matriculation  difficulty | .85 | 4.90 | .000 | .597 | 3.17 | .002 | .60 | 3.16 | .002 |
| Psychometric score | 1.60 | 9.63 | .000 | 1.55 | 9.34 | .000 | 1.55 | 9.35 | .000 |
| Attachment vs. Neutral primes |  |  |  | .06 | .16 | .876 | .02 | .05 | .964 |
| Positive vs. Neutral primes |  |  |  | -.17 | -.47 | .636 | -.19 | -.50 | .616 |
| Math identification (MI) |  |  |  | .61 | 3.30 | .001 | .42 | 1.60 | .111 |
| Attachment X MI |  |  |  |  |  |  | .70 | 1.87 | .062 |
| Positive X MI |  |  |  |  |  |  | .01 | .04 | .972 |
| *Note*. *N* = 474 female students.  Model summary Block I: *R* = .55, *R^2^ =* .30, Δ*R^2^=* .30, *R^2^_ad_*_j_ = .29, *F*(3,470) = 66.74, *p* < .001, Δ*F* = 66.74, *p* < .001.  Model summary Block II: *R* = .56, *R^2^ =* .32*,* Δ*R^2^=*.02, *R^2^_ad_*_j_ = .31, *F*(3,467) = 35.82, *p* < .001, Δ*F* = 3.73, *p* = .011.  Model summary Block III: *R* = .57, *R^2^ =* .32, Δ*R^2^=*.01, *R^2^_ad_*_j_ = .31, *F*(2,465) = 27.52, *p* < .001, Δ*F* = 2.11, *p* = .122. | | | | | | | | | |

| **Supplementary Table** **11**  *Results of Regression Analysis on Math Performance, with the Attachment security condition as the reference category.* | | | | | | | | | |
| --- | --- | --- | --- | --- | --- | --- | --- | --- | --- |
|  | **Block ǀ** | | | **Block ǀǀ** | | | **Block ǀǀǀ** | | |
|  | *B* | *t* | *Sig.* | *B* | *t* | *Sig.* | *B* | *t* | *Sig.* |
| Constant | 8.90 | 58.86 | .000 | 8.99 | 34.46 | .000 | 8.95 | 34.29 | .000 |
| Math related major | .14 | .86 | .391 | -.01 | -.02 | .981 | -.01 | -.07 | .943 |
| Math matriculation  difficulty | .85 | 4.90 | .000 | .60 | 3.17 | .002 | .59 | 3.16 | .002 |
| Psychometric score | 1.60 | 9.63 | .000 | 1.55 | 9.36 | .000 | 1.55 | 9.35 | .000 |
| Positive vs. Attachment primes |  |  |  | -.23 | -.62 | .534 | -.20 | -.54 | .589 |
| Neutral vs. Attachment primes |  |  |  | -.06 | -.16 | .876 | -.02 | -.05 | .964 |
| Math identification (MI) |  |  |  | .61 | 3.29 | .001 | 1.12 | 3.61 | .000 |
| Positive X MI |  |  |  |  |  |  | -.69 | -1.76 | .079 |
| Neutral X MI |  |  |  |  |  |  | -.70 | -1.87 | .062 |
| *Note*. *N* = 474 female students.  Model summary Block I: *R* = .55, *R^2^ =* .30, Δ*R^2^=* .30, *R^2^_ad_*_j_ = .29, *F*(3,470) = 66.74, *p* < .001, Δ*F* = 66.74, *p* < .001.  Model summary Block II: *R* = .56, *R^2^ =* .32*,* Δ*R^2^=*.02, *R^2^_ad_*_j_ = .31, *F*(3,467) = 35.82, *p* < .001, Δ*F* = 3.73, *p* = .011.  Model summary Block III: *R* = .57, *R^2^ =* .32, Δ*R^2^=*.01, *R^2^_ad_*_j_ = .31, *F*(2,465) = 27.52, *p* < .001, Δ*F* = 2.11, *p* = .122. | | | | | | | | | |

| **Supplementary Table** **12**  *Results of Regression Analysis on Math Performance, with the Attachment security condition as the reference category.* | | | | | | | | | | | |
| --- | --- | --- | --- | --- | --- | --- | --- | --- | --- | --- | --- |
|  | **Block ǀ** | | | **Block ǀǀ** | | | | **Block ǀǀǀ** | | | |
|  | *B* | *t* | *95% CI* | *B* | *t* | *95% CI* | | *B* | *t* | *95% CI* | |
| Constant | 8.90 | 58.82*** | [8.61,9.20] | 8.85 | 47.94*** | | [8.49,9.21] | 8.84 | 34.29*** | | [8.48,9.20] |
| Math related major | .15 | .87 | [-.18,.47] | .006 | .04 | [-.31,.34] | | -.002 | -.01 | [.34,.33] | |
| Math matriculation  difficulty | .86 | 4.95*** | [.52,1.20] | .61 | 3.23** | [.24,.98] | | .61 | 3.23** | [.24,.98] | |
| Psychometric score | 1.61 | 9.67*** | [1.28,1.94] | 1.56 | 9.40*** | [1.23,1.89] | | 1.56 | 9.42*** | [1.23,1.88] | |
| ECR Anxiety | .17 | 1.12 | [-.13,.48] | .15 | .95 | [-.16,.45] | | .16 | 1.07 | [-.14,.47] | |
| ECR Avoidance | .04 | .23 | [-.27,.34] | .01 | .07 | [-.29,.31] | | -.01 | -.06 | [-.31,.29] | |
| Math identification (MI) |  |  |  | .59 | 3.20** | [.23,.96] | | .407 | 1.99* | [.004,.81] | |
| Attachment v. Other primes |  |  |  | .17 | .54 | [-.46,.81] | | .15 | .47 | [-.48,.78] | |
| Identification X Attachment |  |  |  |  |  |  | | .71 | 2.09* | [.04,1.38] | |

Note. N = 474 female students. * p < .05, ** p < .01, *** p < .001

Model summary Block I: R = .55, R2 = .30, ΔR2= .30, R2adj = .29, F(5,468) = 40.28, p < .001, ΔF = 40.28, p < .001.

Model summary Block II: R = .56, R2 = .32, ΔR2=.02, R2adj = .31, F(7,466) = 30.79, p < .001, ΔF = 5.23, p = .006.

Model summary Block III: R = .57, R2 = .32, ΔR2=.01, R2adj = .31, F(8,465) = 27.69, p < .001, ΔF = 4.38, p = .037.

**Supplementary Table** **13**

*Mean and standard deviations for the emotion ratings of each image tested in Study 2*

| Image | Emotion | Mean | SD |
| --- | --- | --- | --- |
| 1 | scared | 1.131579 | 0.414015 |
|  | happy | 2.473684 | 1.006381 |
|  | secure | 1.810811 | 0.995485 |
|  | neutral | 2.394737 | 1.15172 |
| 2 | scared | 1.815789 | 0.865409 |
|  | happy | 1.131579 | 0.577556 |
|  | secure | 1.297297 | 0.740303 |
|  | neutral | 2.368421 | 1.125168 |
| 3 | scared | 1.342105 | 0.627148 |
|  | happy | 2.052632 | 0.984951 |
|  | secure | 1.621622 | 0.758337 |
|  | neutral | 2.052632 | 0.836575 |
| 4 | scared | 1.184211 | 0.392859 |
|  | happy | 1.052632 | 0.226294 |
|  | secure | 1.243243 | 0.494717 |
|  | neutral | 2.368421 | 1.261082 |
| 5 | scared | 1.052632 | 0.226294 |
|  | happy | 1.842105 | 1.000711 |
|  | secure | 1.540541 | 0.767195 |
|  | neutral | 2.105263 | 1.157571 |
| 6 | scared | 1.105263 | 0.388307 |
|  | happy | 1.684211 | 1.016227 |
|  | secure | 1.472222 | 0.774084 |
|  | neutral | 2.210526 | 1.14273 |

**Supplementary Table** **14**

| *Ethnicity* | | | |
| --- | --- | --- | --- |
|  | | N | % |
| African-American | | 28 | 3.4% |
| Caucasian | | 525 | 63.4% |
| Asain | | 74 | 8.9% |
| Indian | | 13 | 1.6% |
| Middle Eastern | | 26 | 3.1% |
| Latino/Hispanic | | 79 | 9.5% |
| Other | | 70 | 8.5% |
| Prefer not to answer | | 12 | 1.4% |
| Total |  | 827 |  |

**Supplementary Table** **15**

| *Tests of Between-Subjects Effects on Domain ID by condition* | | | | | | |
| --- | --- | --- | --- | --- | --- | --- |
| Source | Type III Sum of Squares | df | Mean Square | F | Sig. | Partial Eta Squared |
| Corrected Model | 1.165^a^ | 3 | .388 | .250 | .862 | .001 |
| Intercept | 7913.805 | 1 | 7913.805 | 5092.265 | <.001 | .842 |
| Prime Condition | .095 | 1 | .095 | .061 | .805 | .000 |
| Math Condition | .245 | 1 | .245 | .158 | .692 | .000 |
| Prime Condition * Math Condition | .848 | 1 | .848 | .545 | .460 | .001 |
| Error | 1482.596 | 954 | 1.554 |  |  |  |
| Total | 9403.667 | 958 |  |  |  |  |
| Corrected Total | 1483.760 | 957 |  |  |  |  |
| *Note*: R Squared = .001 (Adjusted R Squared = -.002). Prime condition [neutral, attachment security priming], Math condition [no threat, stereotype threat]. | | | | | | |

**Supplementary Table** **16**

| *Condition * Major Crosstabulation* | | | | |
| --- | --- | --- | --- | --- |
|  | | Major | | Total |
|  |  | Non-STEM | STEM |  |
| condition | Neutral-No Threat | 110 | 92 | 202 |
|  | Secure-No Threat | 124 | 88 | 212 |
|  | Neutral-Threat | 105 | 99 | 204 |
|  | Secure- Threat | 120 | 90 | 210 |
| Total | | 459 | 369 | 828 |

X^2^(3)= 2.425, P = 0.489, Cramer’s V = 0.054

**Supplementary Table** **17**

*ANOVA Summary Table for Study 2*

___________________________________________________________________________

Source *df* MS *F p* Effect Size

____________________________________________________________________________________

Education 1 12.605 3.288 .070 .005

Math Grade 1 61.872 16.139 .<.001 .023

Major 1 34.069 8.887 <.003 .013

Domain ID 1 69.257 18.065 <.001 .026

Prime 1 .082 .021 .884 .000

Threat 1 .302 .079 .779 .000

Major x Domain ID 1 2.093 .546 .460 .001

Major x Prime 1 2.699 .701 .403 .001

Major x Threat 1 .335 .087 .768 .000

Domain ID x Prime 1 .130 .034 .854 .000

Domain ID x Threat 1 2.093 .546 .460 .001

Prime x Threat 1 1.394 .364 .547 .001

Major x Domain ID x Prime 1 .156 .041 .840 .000

Major x Domain ID x Threat 1 1.090 .284 .594 .000

Major x Prime x Threat 1 .559 .146 .703 .000

Domain ID x Prime x Threat 1 1.593 .416 .519 .001

Threat x Prime x 1 12.105 3.157 .076 .005

Domain ID x Major

Within groups 811 4.154

Total 827

_____________________________________________________________________*Note.* MS = Mean squares, effect size = *η2* or partial *η2*. Independent variables were priming condition [neutral, secure], threat condition [no threat, threat], math domain identification [low, high], and major [non-STEM, STEM].

1. ^1^ The preregistered analysis included only prime, threat, and domain identification (Major and Domain ID). The covariates education and self-reported average high school math grade markedly changed the p-value of the four-way interaction. The analysis was changed to exploratory to accommodate the covariates not included in the preregistration. [↑](#footnote-ref-1)
