# Supplementary material for: Effects of attachment security priming on women’s math performance
Source: Front Psychol. 2023 Aug 24;14:1124308. doi: 10.3389/fpsyg.2023.1124308 (PMC10484519; doi:10.3389/fpsyg.2023.1124308)
Supplement: Supplementary file 2 [file Data_Sheet_1.docx]

**Supplementary
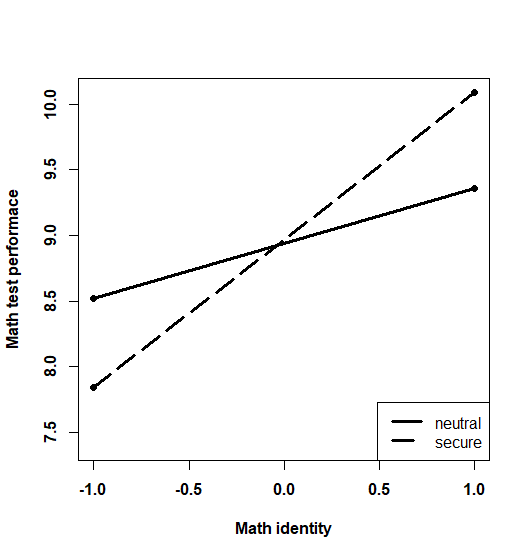
Figure 1**

The interaction effect of exposure to attachment security primes (vs. neutral primes) and math identification (*N* = 474) on math test performance among women, *B* = 0.70 (*SE* = 0.37), *t* = 1.87, *p* = .062.
