# Supplementary material for: Effects of attachment security priming on women’s math performance
Source: Front Psychol. 2023 Aug 24;14:1124308. doi: 10.3389/fpsyg.2023.1124308 (PMC10484519; doi:10.3389/fpsyg.2023.1124308)
Supplement: Supplementary file 3 [file Data_Sheet_2.docx]

**Supplementary
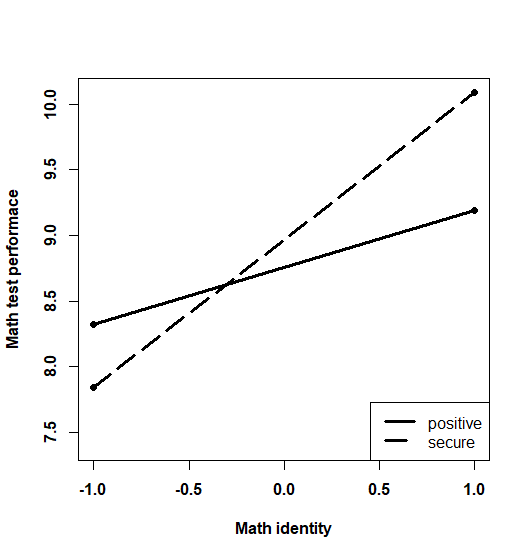
Figure 2**

The interaction effect of exposure to positive prime unrelated to attachment (vs. secure primes) and math identification (*N* = 474) on math test performance among women, *B* = -0.69 (*SE* = 0.39), *t* = 1.76, *p* = .079.
